# Modelling the risk of *Taenia solium* exposure from pork produced in western Kenya

**DOI:** 10.1371/journal.pntd.0005371

**Published:** 2017-02-17

**Authors:** Lian F. Thomas, William A. de Glanville, Elizabeth A. J. Cook, Barend M. De C. Bronsvoort, Ian Handel, Claire N. Wamae, Samuel Kariuki, Eric M. Fèvre

**Affiliations:** 1 University of Edinburgh, Centre for Infection, Immunology and Evolution, School of Biological Sciences, Ashworth Laboratories, West Mains Rd, Edinburgh, Scotland; 2 International Livestock Research Institute, Nairobi, Kenya; 3 The Roslin Institute and The Royal (Dick) School of Veterinary Studies, University of Edinburgh, Roslin, Midlothian, United Kingdom; 4 Centre for Microbiology Research, Kenya Medical Research Institute, Nairobi, Kenya; 5 Department of Microbiology, School of Medicine, Mount Kenya University, Thika, Kenya; 6 Institute of Infection and Global Health, University of Liverpool, Leahurst Campus, Neston, United Kingdom; Texas A&M University College Station, UNITED STATES

## Abstract

The tapeworm *Taenia solium* is the parasite responsible for neurocysticercosis, a neglected tropical disease of public health importance, thought to cause approximately 1/3 of epilepsy cases across endemic regions. The consumption of undercooked infected pork perpetuates the parasite’s life-cycle through the establishment of adult tapeworm infections in the community. Reducing the risk associated with pork consumption in the developing world is therefore a public health priority. The aim of this study was to estimate the risk of any one pork meal in western Kenya containing a potentially infective *T*. *solium* cysticercus at the point of consumption, an aspect of the parasite transmission that has not been estimated before. To estimate this, we used a quantitative food chain risk assessment model built in the @RISK add-on to Microsoft Excel. This model indicates that any one pork meal consumed in western Kenya has a 0.006 (99% Uncertainty Interval (U.I). 0.0002–0.0164) probability of containing at least one viable *T*. *solium* cysticercus at the point of consumption and therefore being potentially infectious to humans. This equates to 22,282 (99% U.I. 622–64,134) potentially infective pork meals consumed in the course of one year within Busia District alone. This model indicates a high risk of *T*. *solium* infection associated with pork consumption in western Kenya and the work presented here can be built upon to investigate the efficacy of various mitigation strategies for this locality.

## Author summary

*Taenia solium* is a serious zoonotic helminth which is thought to be responsible for approximately 1/3^rd^ of epilepsy cases in the developing world. The work presented in this paper aimed to understand what the risk is of acquiring *T*. *solium* taeniosis from pork slaughtered and consumed in western Kenya. In order to do this we built a stochastic risk assessment model to investigate the safety of pork reaching the consumer in terms of the risk of having viable *T*. *solium* cysts in any one portion of meat consumed. We found that pork represents a high risk product in this study area and therefore control strategies are urgently needed to reduce the public health risk posed by this product.

## Introduction

The zoonotic tapeworm *Taenia solium*, has a two host life cycle, with humans as the definitive host, and pigs as an intermediate host. Humans are infected after consumption of viable cysticerci in under-cooked pork and harbour the adult tapeworm, a condition known as taeniosis. Gravid proglottids, containing thousands of infective eggs, detach from the adult tapeworm and are excreted in faeces in an intermittent fashion [1]. Ingestion of these eggs, by either pigs or humans, results in the larval stage penetrating the intestinal wall, moving through the lymph and blood vessels to encyst in muscle, eyes or the central nervous system (CNS) as cysticerci [2].

Infection of the central nervous system, neurocysticercosis (NCC), manifests predominately as epileptic seizures and is thought to be responsible for 29.0% (95% C.I. 22.9–35.5%) of epilepsy cases across endemic regions [3]. Due to the highly clustered nature of the parasite the proportion of people with epilepsy (PWE) suffering from NCC will likely be highly variable both between and within individual countries as illustrated by data from Tanzania suggesting that between 7.7% (95% C.I. 1–25) and 23% (95% C.I. 15–31) of epilepsy cases are NCC-associated depending on the geographical location [4]. Understanding the burden of NCC related epilepsy in individual countries requires data on the prevalence of both epilepsy and NCC within those countries, data which is currently lacking in many instances [3, 5]. A recent meta-analysis estimated the overall prevalence of circulating *T*. *solium* antigens in humans of 7.30% (95% CI [4.23–12.31]) for sub-Saharan Africa [6] and it has been estimated that 0.95–3.08 million people in sub-Saharan Africa may suffer from NCC-related epilepsy [7].

*T*. *solium* cysticercosis has been identified as an important disease predominately in Latin America [8], Asia [9] and across much of Africa [6, 10] although the nature of global travel and migration puts individuals from all countries at risk of infection. This is highlighted by cases of NCC being diagnosed in the United States, predominately in immigrants from Latin America with histories indicating that infection was acquired from endemic areas [11–13]. People travelling from endemic areas harbouring *T*. *solium* taeniosis, can in turn expose many other people to *T*. *solium* eggs, who may develop NCC. A well-known example of this was the detection of NCC in members of an Orthodox Jewish community in NYC, the likely source of infection in these cases was believed to be domestic staff originating from endemic areas [14]. In 2012 *T*. *solium* was ranked by FAO as the most important parasitic food safety issue globally [15] and the WHO Foodborne Disease Epidemiology Reference Group (FERG) estimates that it is the foodborne parasite with highest global burden [16]. Better understanding the risk of transmission in the food chain is therefore a priority.

The estimated global burden of cysticercosis has recently been revised, and the parasite is thought to be responsible for a global total of 2,788,426 (95% C.I. 2,137,613–3,606,582) disability adjusted life years (DALYs), annually [16]. The burden of this disease lies disproportionally on developing nations, and even more so those with large rural populations and in which treatment for NCC may be lacking; in Cameroon, for example, the burden was estimated to be 45,838 (95% C.I. 14,108–103,469), equating to 9 DALYs per 1000 person years [5]. It is unclear whether this is due to high NCC-associated mortality in Cameroon compared to that in other countries or represents an over-estimation by the authors. [17]. In Tanzania, it was recently estimated that 0.7 DALYs are lost per 1000 person-years [4] in comparison with an estimate 0.25 DALYs lost per 1000 person-years in Mexico where NCC patients are five times more likely to receive treatment [17]. In Nepal it is estimated that 0.543 (95% C.I. 0.207–1.0543) DALYs are lost per 1000 person years [18]. In the majority of cases, the only NCC-associated sequela considered in DALY calculations to date has been epilepsy, while severe headache was also included in Mexico [4, 5, 17, 18]. Despite the inclusion of headaches in the Mexico study, other manifestations such as: headache, visual disturbances, other signs of increased intra-cranial pressure, cranial nerve palsy, gait abnormality, various focal neurological deficits, altered mental state and pyramidal (upper motor neuron damage) signs [19] have not yet been widely included in DALY calculations due to the lack of good estimates of the proportion of these specific manifestations attributable to NCC. All DALY calculations performed for NCC are therefore likely to be underestimations.

The consumption of undercooked, infected pork is a major risk factor for acquiring taeniosis. Taeniosis, in turn represents an important public health hazard, with an adult *T*. *solium* carrier becoming a focus of infection for both porcine and human cysticercosis [20]. Indeed the consumption of pork [21] and the inability to recognise infected meat [22] are two risk factors which have been significantly associated with human cysticercosis. Other statistically significant risk factors identified in a systematic review from endemic zones (Africa, Latin America and Asia) were; insufficient latrines, history of taeniosis or proximity to carriers, being male and of increased age, lack of potable water, poor personal and house hygiene including washing hands by ‘dipping’, earthen floor, pig owning and/or the presence of infected pigs and low education [6].

Addressing porcine infection and reducing the volume of infected meat entering the food chain is therefore an important issue for public health practitioners in order to reduce the burden of this neglected tropical disease. A process of risk analysis, whereby risks are identified and described, qualitatively and/or quantitatively assessed and then communicated and mitigated, can achieve understanding of the current risks posed by pork consumption in developing countries.

The principal of risk analysis allows scientific, justifiable and transparent decisions to be made regarding the risks associated with food products and is a key component of the Codex Alimentarius framework. Codex Alimentarius is a joint FAO/WHO Commission whose role is to protect consumer safety in its member states in such a way that trade can be conducted in an environment where consistent food safety standards are enforced for all countries, removing the potential for non-tariff barriers to trade [23]. A stochastic, quantitative risk assessment, as part of a risk analysis process, allows us to incorporate quantitative data and the uncertainty and variability that surrounds these data, in order to establish a quantitative estimate of risk and a probability interval around that estimate.

The aim of the work presented here was to estimate the risk to humans of exposure to *T*. *solium* from pork currently entering the food chain in rural western Kenya. We specifically sought to estimate the probability of any one pork meal consumed in western Kenya containing at least one viable, and therefore potentially infective, *T*. *solium* cyst. To this end a stochastic risk assessment model with Monte Carlo simulation was built and informed by data gathered in the field in western Kenya and supplemented by data available in the literature

## Materials and methods

### Ethics

Ethical approval for aspects of field data collection pertaining to humans was granted in March 2010 by the Kenya Medical Research Institute (KEMRI) Ethical Review Committee (SSC No.1701) and all activities were undertaken in accordance with the approved protocols.

Once entered into the study every participant was identified by a unique identifying number and never by name, hence ensuring anonymity of all data. Prior to any data collection each human participant in the study was required to sign, or mark with a thumb print, an informed consent document. This document, including the provision of a thumb print in place of a signature, and its administration by trained staff, was approved by the KEMRI Ethical Review Committee. The steps before signing the informed consent document involved ascertaining the appropriate language for communication, an explanation of the project, the sampling procedure and emphasising that participation was entirely voluntary. One copy of the completed form was retained by the project and one copy was provided to the participant.

Ethical approval for sample collection from animals was granted by the Animal Welfare and Ethical Review Body (AWERB) at The Roslin Institute, University of Edinburgh (approval number AWA004 Bronsvoort). Sampling of privately owned domestic pigs presented to slaughter houses was carried out by trained veterinarians or animal health assistants after obtaining verbal informed consent from the owners of those pigs. Blood sampling from the cranial vena cava was undertaken according to the guidelines provided by the National Centre for the Replacement, Refinement and Reduction of Animals in Research (http://www.nc3rs.org.uk/bloodsamplingmicrosite/page.asp?id=346).

### Risk question

A stochastic risk assessment model was built to answer the following question: “What is the risk that any one pork meal consumed in western Kenya contains at least one viable cysticercus of *Taenia solium*?”

### Risk assessment model

**Fig 1:**
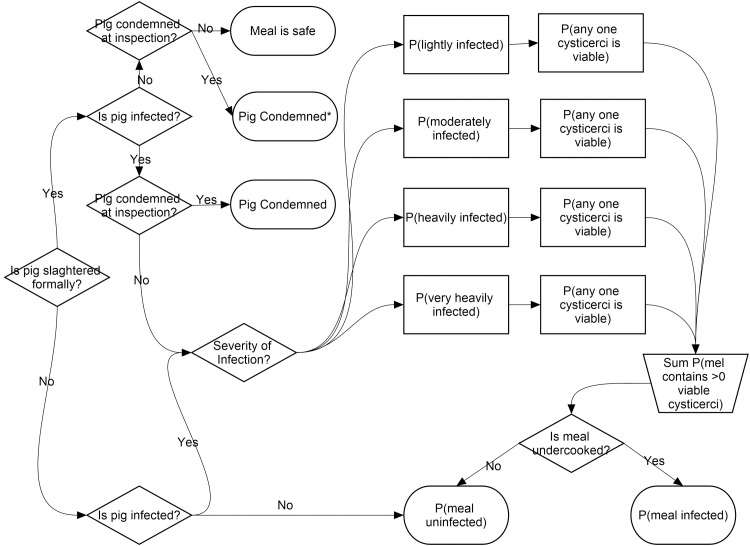
Structure of risk model

A stochastic risk assessment model using Monte Carlo simulation was built using the @Risk (Palisade, Newfield, NY, USA) add-on for Excel (Microsoft corp. USA) which can be found in supporting information S1 and is illustrated in Fig 1.

The probability of any one pork meal being infective at the point of consumption was estimated using a decision tree method which comprehensively included 15 possible field situations, described as conditional probabilities) through which a pork meal could ‘move through’ the food chain from pig to plate. Where pigs are informally slaughtered the probability of the situation is defined as the distribution of infection (including no-infection) conditional on the pig being informally slaughtered. Where the pigs are slaughtered formally the probability of detection of infection at meat inspection is included. The 15 situations are defined as follows;

Situation 1 = Pig is not detected at meat inspection |* pig being lightly infected | Pig is formally slaughtered

Situation 2 = Pig is detected and condemned at meat inspection | lightly infected | Pig is formally slaughtered

Situation 3 = Pig is not detected at meat inspection | moderately infected | Pig is formally slaughtered

Situation 4 = Pig is detected and condemned at meat inspection | moderately infected | Pig is formally slaughtered

Situation 5 = Pig is not detected at meat inspection | heavily infected |Pig is formally slaughtered

Situation 6 = Pig is detected and condemned at meat inspection | heavily infected | Pig is formally slaughtered

Situation 7 = Pig is not detected at meat inspection | very heavily infected |Pig is formally slaughtered

Situation 8 = Pig is detected at meat inspection and condemned | very heavily infected | Pig is formally slaughtered

Situation 9 = Pig is not detected at meat inspection | uninfected | Pig is formally slaughtered

Situation 10 = Pig is detected and condemned at meat inspection (false positive) | uninfected | Pig is formally slaughtered

Situation 11 = Pig is lightly infected | Pig is informally slaughtered

Situation 12 = Pig is moderately infected | Pig is informally slaughtered

Situation 13 = Pig is heavily infected | Pig is informally slaughtered

Situation 14 = Pig is very heavily infected | Pig is informally slaughtered

Situation 15 = Pig is uninfected | Pig is informally slaughtered

* conditional on

With the probability (Pr) of each situation being calculated as;

Pr(situation x) = (Pr (slaughter status)*Pr (Infection status)*Pr (intensity of infection)*Pr (detection status at meat inspection))

The risk of any one pork meal being potentially infective at consumption is expressed as;

Pr(any one pork meal is infective at consumption) = ((Pr(pork meal contains a cyst | Situation 1)*Pr(Situation 1) + Pr(pork meal contains a cyst | Situation 2)*Pr(Situation 2)+ Pr(pork meal contains a cyst | Situation 3)*Pr(Situation 3)…… + Pr(pork meal contains a cyst | Situation 15)*Pr(Situation 15))*Pr(any one cyst is viable prior to cooking))* Pr(Meal undercooked)

Briefly this illustrates that the overall probability that any one pork meal is infective at the point of consumption is calculated through the probability of a meal containing a cyst (viable or degraded) given any of the 15 situations described is multiplied by the probability of that situation. These calculations are repeated for each of the 15 situations. The sum of these probabilities is then multiplied by the probability that any one cyst in any meal is viable prior to cooking to give the probability of any one pork meal being infective prior to cooking. This probability is then multiplied again by the probability of a meal being undercooked in the western Kenya context to give an overall probability of any one pork meal being infective at the point of consumption.

We used the ‘Auto’ function in @Risk, a function which runs sufficient iterations, to a maximum of 50,000, until all input parameters have converged, using the default settings of 3% tolerance and 95% confidence, i.e. when there is a 95% probability that the mean of the tested output is within +_/-_3% of its “true” expected value, based upon the accumulated data from the iterations already run.

To estimate the total number of potentially infective pork meals consumed in the course of one year within Busia District the following equation was included in the model:

Number of potentially infective pork meals consumed per annum (Busia County) = Pr(meal infective) * (number of pork meals consumed per year)

Where:

Number of pork meals consumed per year = (number of people consuming pork daily *365) + (number of people consuming pork weekly *52) + (number of people consuming pork monthly*12) + (number of people consuming pork yearly *1) + (number of people consuming pork on special occasions *0.5)

Where the number of people consuming pork (daily/weekly/monthly/yearly/special occasions) = population of Busia county (Model parameter P20) * proportion of population consuming pork (daily/weekly/monthly/yearly or on special occasions) (Model parameter P21-25).

### Model parameters

The parameters of each model input are described fully in Table 1. Beta-PERT distributions were used for the prevalence of porcine cysticercosis in the porcine population, the proportion of pigs slaughtered informally, the proportion of pigs suffering from infections of varying intensity and the proportion of pork meals eaten undercooked. Beta-PERT distributions have been recommended for providing a natural distribution from expert opinion of the minimum, maximum and most likely values of an input and are bound between 0 and 1 [24]. PERT stands for ‘Program Evaluation and Review Technique’ and was a distribution first used for assessing the development schedule and costs of the Polaris weapons system [25]. The distribution is unimodal, continuous and has two non-negative x-axis intercepts which make it suitable for the data being modelled in this study [26]. The distribution was determined using the ‘Beta-PERT’ function, method = ‘Vose’ in the package “Prevalence” [27] for R [28]. The Beta-PERT methodology allows one to parametrize a generalized Beta distribution based on expert opinion regarding a pessimistic estimate (minimum value), a most likely estimate (mode), and an optimistic estimate (maximum value). The maximum and minimum limits of the distributions were set using the 99.9% confidence intervals from field data as we felt that ‘true life’ data from the field would be a more accurate reflection of reality than an ‘expert’ opinion. Uniform distributions were used for the probability of any one pork meal containing a cyst and the probability of any one cysticercus being viable reflecting the high degree of uncertainty surrounding these values.

**Table 1:** Description of model parameters.

Parameter	Description	Source	Probability (99.9% C.I.)	Distribution(α,β)
P1	Probability pig was slaughtered informally	3 out of 69 pig owning homesteads practice home slaughter [38]	0.043 (0.002–0.187)	Beta-PERT(1.89,4.11)
P2	Probability pig is infected (formal slaughter)	Prevalence of cysticercosis as detected by HP10 Antigen ELISA adjusted for diagnostic test parameters (Se 89.5% (95% CI. 82.3–94.2%), Sp 74% (95% CI. 56.6–87.6) [30] [39]	0.376 (0.238–0.513)	Beta-PERT(3.01,2.99)
P3	Probability pig is infected (informal slaughter)	Literature indicates no significant difference between prevalence at formal and informal slaughter [40]	0.376 (0.238–0.513)	Beta-PERT(3.01,2.99)
P4	Probability pig is lightly infected (<50 cysts)*	Enumeration of cysts in 31 pigs from Zambia assessed by carcass dissection. From a random selection of 65 pigs Light (15/31) Mod (2/31) Heavy (12/31) [41] Heavy infections then broken into heavy and very heavy infection on the assumption that lingual palpation detects only very heavily infected animals [30, 41–45]. Therefore using the sensitivity of lingual palpation, estimated to be 16.1% (95% C.I. 5–34%), [43] as a proxy for the proportion of very heavy infection. indicating 2/31 pigs would fall into the ‘very heavy category’ [41]	0.484 (0.206–0.770)	Beta-PERT(2.87,3.03)
P5	Probability pig is moderately infected (50>100 cysts)*		0.065 (0.001–0.331)	Beta-PERT(1.78,4.22)
P6	Probability pig is heavily infected (100>500 cysts)*		0.387 (0.137–0.689)	Beta-PERT(2.78,3.22)
P7	Probability pig is very heavily infected (>500 cysts)*		0.065 (0.001–0.331)	Beta-PERT(1.78,4.22)
P8	Probability infected pig is detected at meat inspection (as currently performed in the study area)	Meat inspectors reported no condemnation of carcasses for any reason during the course of a field survey [39]	0 (0–0.068)	Beta-PERT(1,5)
P9	Probability uninfected pig is detected at meat inspection (false positive)		0 (0–0.048)	Beta-PERT(1,5)
P10	Mean number of meals per pig	Mean dressed weight/pig 22.5kg [46] Assumption of 100g/person mean portion size		225**
P11	Probability any one meal contains at least one cyst (lightly infected pig)	1–50 cysts per pig/mean number of meals per pig based on dissection of the musculature of 6 pigs[47]		Uniform(1,50)
P12	Probability any one meal contains at least one cyst (moderately infected pig)	51–100 cysts per pig/mean number of meals per pig based on dissection of the musculature of 2 pigs[45, 47]		Uniform(51,100)
P13	Probability any one meal contains at least one cyst (heavily infected pig)	101–500 cysts per pig/mean number of meals per pig based on dissection of the musculature of 3 pigs [47]		Uniform(101,500)
P14	Probability any one meal contains at least one cyst (very heavily infected pig)	501–80340 cysts per pig/mean number of meals per pig based on dissection of the musculature of 23 pigs [45]		Uniform(501,80340)
P15	Probability any one meal contains at least one cysticercus (uninfected pig)	No cysticercus present		0**
P16	Probability any one meal contains at least one cysticercus (pig detected at meat inspection and condemned	No carcass present		0**
P17	Probability any one cysticercus is viable	Proportion of viable cysticercus in carcasses from 1% to 100% [48, 49]		Uniform(0.01,1)
P18	Probability pork eaten undercooked	98/1386 pork eaters expressed preference for undercooked pork [38]	7.07 (5.01–9.61)	Beta-PERT(2.80,3.20)
P19	Number of pigs slaughtered in Busia District/Year	21,315 pigs in Busia District [34] Assumption of a complete turnover of pig population each year Minus a 20% crude mortality in smallholder grower-finisher systems in Kenya [50]		21,315**
P20	Estimated population of Busia County	230,253 [30] of which 76.0% report consuming pork [34]		230,253**
P21	Proportion of Busia population consuming pork daily	15/2116 people reported consuming pork daily [38]	0.007 (0.004–0.014)	Beta-PERT(2,2, 3.8)
P22	Proportion of Busia population consuming pork weekly	345/2116 people reported consuming pork weekly [38]	0.164 (0.143–0.185)	Beta-PERT(3,3)
P23	Proportion of Busia population consuming pork monthly	808/2116 people reported consuming pork monthly [38]	0.382 (0.355–0.409)	Beta-PERT(0.5,0.5)
P24	Proportion of Busia population consuming pork yearly	347/2116 people reported consuming pork yearly [38]	0.164 (0.144–0.186)	Beta-PERT(2.9, 3.1)
P25	Proportion of Busia population consuming pork on special occasions	9/21167 people reported consuming pork only on special occasions (we assume here an average of once every 2 years) [38]	0.046 (0.035–0.059)	Beta-PERT(2.8, 3.1)

*Probabilities scaled (Probability of infection intensity/sum of all infection intensity probabilities) so that no iteration can sum to a probability >1

**fixed variable.

### Sources of data

Each parameter in the model was informed either by field data or from the literature where field data were lacking. Literature used is referenced against the appropriate parameter in Table 1.

The Kenyan specific field data were obtained from two complementary studies, both undertaken in the same area of western Kenya, which is representative of the Lake Victoria Basin ecosystem. The first study was a community based cross-sectional study of humans and their livestock from 416 randomly selected homesteads between July 2010 and July 2012, during which questionnaire data were collected on a wide range of homestead and individual level risk factors for zoonotic disease, including meat preparation [29].

The second study investigated the prevalence of *T*. *solium* cysticercosis in 343 pigs slaughtered at registered slaughter premises within the same study site[39]. This study used the HP10 Antigen-ELISA which detects circulating antigen from the parasite with an estimated sensitivity of 89.5% (95% C.I. 82.3–94.2%) and specificity of 74% (95% C.I. 56.6–87.6%)[30]. Apparent prevalence of porcine cysticercosis was used to estimate the true prevalence after adjustment for an imperfect test using the ‘epi.prev’ function. This function uses apparent prevalence, test sensitivity and test specificity to estimate true prevalence. Confidence intervals for all other variables were determined using the ‘epi.conf’ function, which calculates the confidence interval for proportions using the method first proposed by Wilson [31]. Both functions are found in the package ‘EpiR’ [32] within the ‘R’ environment for statistical computing [33].

### Sensitivity analysis

A sensitivity analysis was performed to determine the influence of the input parameters on the main output; the probability that pork meal contains at least one viable cysticercus at consumption. The sensitivity analysis was performed in two stages. Spearman rank order correlation coefficients (ρ values) were calculated and a tornado graph produced. This illustrates the relationship between each input and the output of interest, with ρ values near 0 illustrating the input has little effect on the output through to a value at -1 or +1 illustrating that the output is fully dependent on this input. Key inputs (those with ρ >0.1) were then selected to include in an advanced sensitivity analysis.

An advanced sensitivity analysis was then performed with 35simulations of 500 iterations, monitoring the effect of a range of n^th^ percentiles (1%, 5%, 25%, 50%, 75%, 95%, 99%) of the probability distributions of each selected input on the mean of the outcome. A sensitivity tornado graph was plotted illustrating the effect of the key inputs on the mean of the output.

## Results

### Estimated current infection risk from pork consumed in western Kenya

**Fig 2:**
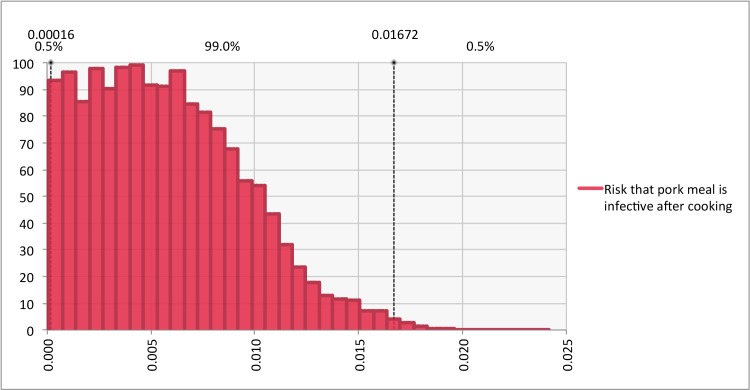
Relative frequency histogram illustrating risk of any one pork meal being infected with a viable *T*. *solium* cysticercus at consumption

After 5,600 iterations all parameters in the model had converged. The model predicted that under the current conditions, any one pork meal consumed (after cooking) in western Kenya has a probability of 0.006 (99% Uncertainty Interval (U.I). 0.0002–0.0164) of containing at least one viable *T*. *solium* cysticercus, and therefore being potentially infective to humans (Fig 2). This equates to 22,282 (99% U.I. 622–64,134 potentially infective pork meals consumed in the course of one year within Busia District alone with a human population of 230,253 [34]. Meat inspection, as is currently practised in western Kenya is responsible, according to the model, for avoiding only 1,397 (99% U.I. 5–8,368) potentially infective meals a year. The probability of each of the 15 situations described within the model (e.g. Situation 1 = Pig is not detected at meat inspection | lightly infected | Pig is formally slaughtered etc) is reported in Table 2.

**Table 2:** Probabilities of each situation described in the model.

Situation	Probability	99% Uncertainty Interval
Situation 1 = Pig is not detected at meat inspection | lightly infected | Pig is formally slaughtered	0.172	(0.078–0.29)
Situation 2 = Pig is detected and condemned at meat inspection | lightly infected | Pig is formally slaughtered	0.002	(0.00001–0.0087)
Situation 3 = Pig is not detected at meat inspection | moderately infected | Pig is formally slaughtered	0.023	(0.002–0.101)
Situation 4 = Pig is detected and condemned at meat inspection | moderately infected | Pig is formally slaughtered	0.000	(0.000001–0.0026)
Situation 5 = Pig is not detected at meat inspection | heavily infected |Pig is formally slaughtered	0.138	(0.0545–0.243)
Situation 6 = Pig is detected and condemned at meat inspection | heavily infected | Pig is formally slaughtered	0.002	(0.00001–0.0074)
Situation 7 = Pig is not detected at meat inspection | very heavily infected |Pig is formally slaughtered	0.023	(0.002–0.099)
Situation 8 = Pig is detected at meat inspection and condemned | very heavily infected | Pig is formally slaughtered	0.000	(0.000001–0.0024)
Situation 9 = Pig is not detected at meat inspection | uninfected | Pig is formally slaughtered	0.592	(0.4620–0.7072)
Situation 10 = Pig is detected and condemned at meat inspection (false positive) | uninfected | Pig is formally slaughtered	0.005	(0.00003–0.01873)
Situation 11 = Pig is lightly infected | Pig is informally slaughtered	0.008	(0.00087–0.0347)
Situation 12 = Pig is moderately infected | Pig is informally slaughtered	0.001	(0.00007–0.0098)
Situation 13 = Pig is heavily infected | Pig is informally slaughtered	0.006	(0.0007–0.029)
Situation 14 = Pig is very heavily infected | Pig is informally slaughtered	0.001	(0.00007–0.0103)
Situation 15 = Pig is uninfected | Pig is informally slaughtered	0.027	(0.0034–0.0971)
Sum Situation Probabilities	1.000	

### Sensitivity analysis

**Fig 3:**
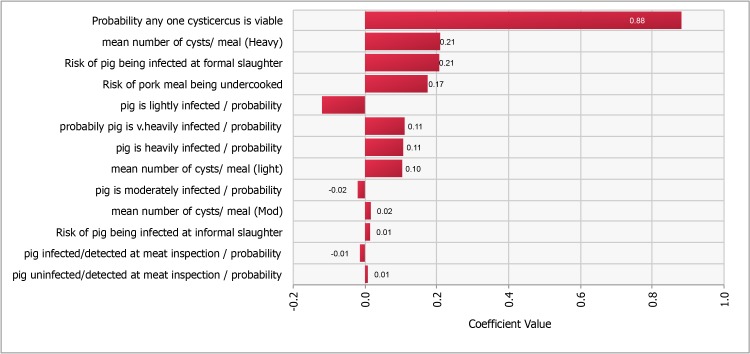
Tornado Graph illustrating the Spearman’s rank order correlation co-efficient values for different inputs.

A tornado graph illustrating the Spearman rank order correlation coefficients can be seen in Fig 3.

The most influential input was the probability that any one cysticercus is viable (ρ = 0.91), followed by the probability a pig is infected at formally slaughtered (ρ = 0.16). The 5 inputs with (ρ >0.1) were selected for inclusion in the advanced sensitivity analysis. This analysis illustrates the range of potential outputs which could be produced by this model based upon the cumulative probability of the selected input distributions.

**Fig 4:**
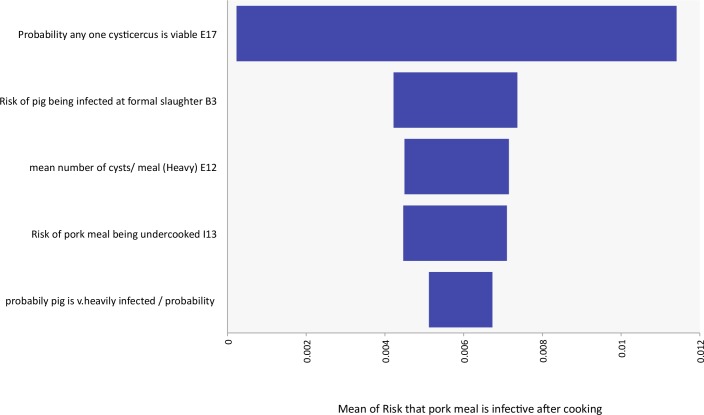
Sensitivity Tornado Graph illustrating change to the mean (Probability any one meal contains a viable cyst after cooking) related to changes in percentiles of input distributions

The analysis suggested that with all other parameters fixed, the probability that any one cysticercus is viable (prior to cooking) has the largest effect on the mean output, from a probability of a meal consumed containing a viable cysticercus of 0.0002 when the input was fixed at the 1^st^ percentile to a probability of 0.011 when the input was fixed at the 99^th^ percentile. The effect upon the outcome of fixing the most influential 5 input variables monitored in the sensitivity analysis at the 1^st^ and 99^th^ percentile can be found in Table 3 and expressed graphically in Fig 4. The full sensitivity analysis report can be found in supporting information S1.

**Table 3:** Influence of changes in input parameters from 1^st^ to 99^th^ percentile of probability distribution upon the mean output probability.

Input parameter (ρ)	Mean probability a pork meal contains at least one viable cysticercus at consumption	
	1^st^ Percentile of input distribution	99^th^ Percentile of input distribution
Probability any one cysticercus was viable (prior to cooking) (0.88)	0.0002	0.013
Mean number of cysts/meal (heavy infection) (0.21)	0.004	0.007
Probability a pig is infected at formal slaughter (0.20)	0.004	0.007
Probability pork eaten undercooked (0.17)	0.005	0.007
Probability that pig was very heavily infected (0.11)	0.005	0.007

## Discussion

This stochastic risk model has enabled us to express, quantitatively, the risk that pork entering the food chain in western Kenya poses to consumers in terms of potential for infection with the zoonotic tapeworm *T*. *solium*. It allows us to better understand the risk of exposure to *T*. *solium* in this setting and provides a tool with which the impact and cost-effectiveness of potential mitigation strategies can be explored. We have attempted to build a simple model using transparent parameters and drawn from either our own field data or published literature.

Pork consumed in western Kenya presents a risk to consumers of exposure to *T*. *solium*. With the current input parameters, there is 0.006 (99% U.I. 0.0002–0.0167) probability that any one pork meal consumed in western Kenya is infected with a viable *T*. *solium* cysticercus, and is therefore potentially infectious to humans. This equates to approximately 22,000 potentially infectious meals being consumed in Busia district alone in any one year, among a human population of over 230,000. It must be noted however, that not each potentially infectious meal consumed will lead to a case of taeniosis. The probability of infection after exposure is not yet understood and may not be high, if we consider the generally low prevalence of *T*. *solium* taeniosis even in areas known to be endemic for porcine cysticercosis [35].

It is known that the risk of acquiring NCC is increased not only in those who have a history of an adult *T*. *solium* infection [36] but also in those living within the vicinity of a taeniosis case [13, 20] or coming into contact with infective eggs through food prepared by a *T*. *solium* carrier who fails to adhere to good hygiene practices. The implication of this is that any one person acquiring an adult *T*. *solium* infection has the potential to expose many more people to the parasite, putting them at risk of NCC. Moreover, individuals with taeniosis provide a source of infective material that can be consumed by pigs, propagating the parasitic life-cycle. Consumption of infectious pork products therefore not only puts the consumer at risk for NCC, but also people around them, therefore it is imperative that the consumption of pork containing viable cysticerci is prevented. There are several strategies that have been suggested for the control of *T*. *solium* although evidence for their efficacy is still scarce [37]. Work is ongoing to investigate the cost-effectiveness of several of these strategies in reducing infection risk utilising the model described here.

While the input parameters for this model were defined using the best data available at the time, it is important to be explicit about some of the assumptions made. One key assumption relates to the probability of any one meal containing a viable cyst within the different categories of infection intensity. It was assumed in this analysis that cysticerci are distributed evenly throughout the musculature of a pig and therefore the probability of any one meal containing a cyst was calculated by dividing the range of cyst numbers for each infection category by the average number of ‘meals’ that one pig can produce (in terms of kg of meat). The reality is more likely to reflect a more un-even distribution of cysts throughout a carcass, but the ability to model this was beyond the scope of the data used in this analysis. The proportion of pigs falling into each infection intensity category was based upon the results of a random selection of pigs from Zambia (Southern and Eastern Provinces) and we cannot be sure that this can be translated to this Kenyan population. We do know, however, that these pigs were randomly selected from a population of slaughter age pigs (1–5 years) in an endemic sub-Saharan country and we therefore have no reason to believe the infection intensity proportions should be different. The probability of any one cysticercus being viable (prior to cooking) was identified as being the most influential variable in this model through the sensitivity analysis and this is predominately due to the wide distribution used to parameterise the variable. The variable was informed by two studies which indicated a large range (1–100%) of viable cysts in carcasses. There is as yet no data which could be used to better inform this distribution and although dissection of larger numbers of pigs may help, differences between pigs in time of age at exposure or in host-parasite immune-response, mean it may be very difficult to produce a more precise estimate. The results of this model should therefore be considered with these assumptions in mind.

A quantitative risk assessment such as that presented here provides a transparent and reproducible way of assessing the current state of risk from a food product. The presence of *T*. *solium* in the porcine population combined with the poor risk mitigation shown by the pork industry as presently structured in western Kenya poses a significant public health hazard and requires a concerted effort by policy makers and other stakeholders to address.

## Supporting information

S1 Model Risk Analysis Model. (XLSX)

S1 Sensitivity Analysis Output of Sensitivity Analysis for Risk Analysis Model. (XLSX)
